# Geographical Detector-Based Identification of the Impact of Major Determinants on Aeolian Desertification Risk

**DOI:** 10.1371/journal.pone.0151331

**Published:** 2016-03-17

**Authors:** Ziqiang Du, Xiaoming Xu, Hong Zhang, Zhitao Wu, Yong Liu

**Affiliations:** 1 Institute of Loess Plateau, Shanxi University, Taiyuan, Shanxi, China; 2 College of Environmental & Resource Science, Shanxi University, Taiyuan, China; Centro de Investigacion Cientifica y Educacion Superior de Ensenada, MEXICO

## Abstract

Arid and semi-arid areas in North China are facing the challenge of a rising aeolian desertification risk (ADR) due to the intertwined effects of complex natural processes and intensified anthropogenic activities. An accurate quantitative assessment of the relationship between ADR and its determinants is beneficial for understanding the driving mechanisms of aeolian desertification and for controlling future desertification. Previous studies have failed to quantify the relative role of determinants driving ADR and have been limited in assessing their interactive impacts. In this study, a spatial variance analysis-based geographical detector methodology is used to quantify the effects of geological, physical, and human factors on the occurrence of ADR in an area characterized by mountains and hills in northern China. It is found that soil type, precipitation, and wind velocity are the major determinants of ADR, which implies that geological and physical elements (e.g., soil attribute) and climatic factors (e.g., precipitation and wind velocity) rather than human activities have played a greater role in the incidence of ADR. Particularly, the results show that the interaction of various determinants causes significant non-linearly enhanced impacts on the ADR. The findings of our study will assist local inhabitants and policy makers in developing measures for wind prevention and sand control to mitigate the effects of desertification in the region.

## Introduction

According to the 1998 United Nations Convention to Combat Desertification (UNCCD), desertification is defined as land degradation in arid, semi-arid, and dry sub-humid areas due to a variety of factors, particularly climatic variations and human activities[1]. A relatively rapid pace of desertification has been observed in many regions around the world. Until today, nearly 1.3 billion people, living in more than 110 countries have suffered from adverse effects of desertification [2]. In Northern China, the main form of desertification is referred to as aeolian desertification, in contrast to other land degradation processes (e.g., water erosion and salinization) [3, 4]. Aeolian desertification is frequently accompanied by surfaces partially or entirely covered by loose sand and finer particles. According to the China National Committee for the Implementation of the UN Convention to Combat Desertification, the total desertification area in China has reached a size of up to 2.622 million km^2^.

Both natural and anthropogenic factors can cause desertification, which can be highly heterogeneous in both spatial and temporal scales. Accordingly, it is challenging, yet urgent to quantify the driving factors of desertification [5]. As mentioned above, driving forces of desertification involve climate variations and unsustainable human activities. For instance, some studies have indicated adverse climatic effects (e.g., drought, severe wind erosion, temperature fluctuation, and winter precipitation) as the primary causes of desertification [6, 7]. Other researchers have attributed desertification to improper agricultural practices (e.g., long-term overgrazing, extensive clearcutting, and widespread conversion of grassland to cropland) [8, 9]. However, no consensus has been reached regarding the exact role of climate versus human activities. Although recent studies have investigated the relative role of climate dynamics and human activities in desertification [10, 11], they have not quantitatively determined the relative importance of each specific driving factor (e.g., soil texture, population, precipitation, etc.), nor have they addressed the joint impacts of these factors on desertification. Furthermore, previous results were not suitable for policy-making or further scientific investigations because of the coarse resolution of these studies [12]. Therefore, there is an urgent need for more detailed studies of the determinants of desertification.

Previous studies have used various methods to quantify the driving forces behind desertification, including regression analysis, factor analysis, principal components analysis, and multiple variable analysis [13–15]. However, the abovementioned conventional statistical approaches are inadequate to analyze dynamic desertification phenomena [16]. In addition, these methods cannot handle the dimensional match between coarsely resolved regional-scale socioeconomic data and more finely resolved raster-scale desertification data [17]. A geographical detectors model proposed by Wang et al [18] and based on spatial variance analysis, has been widely used to measure the association between environmental risks (e.g., earthquake-based mortality and infectious disease) and their determinants [18–21]. This method has made it possible to analyze the driving factors behind desertification [22].

The article attempts to answer the following questions: (1) which environmental parameter is the principal determinant of aeolian desertification risk (ADR)? (2) What is the relative importance of a single determinant of ADR? (3) Do the factors influencing ADR work independently or in combination with each other? This study is instrumental in providing a basic understanding of the role determinants play in triggering ADR and the interactions between these determinants.

## Data and Methods

### Study area

**Fig 1:**
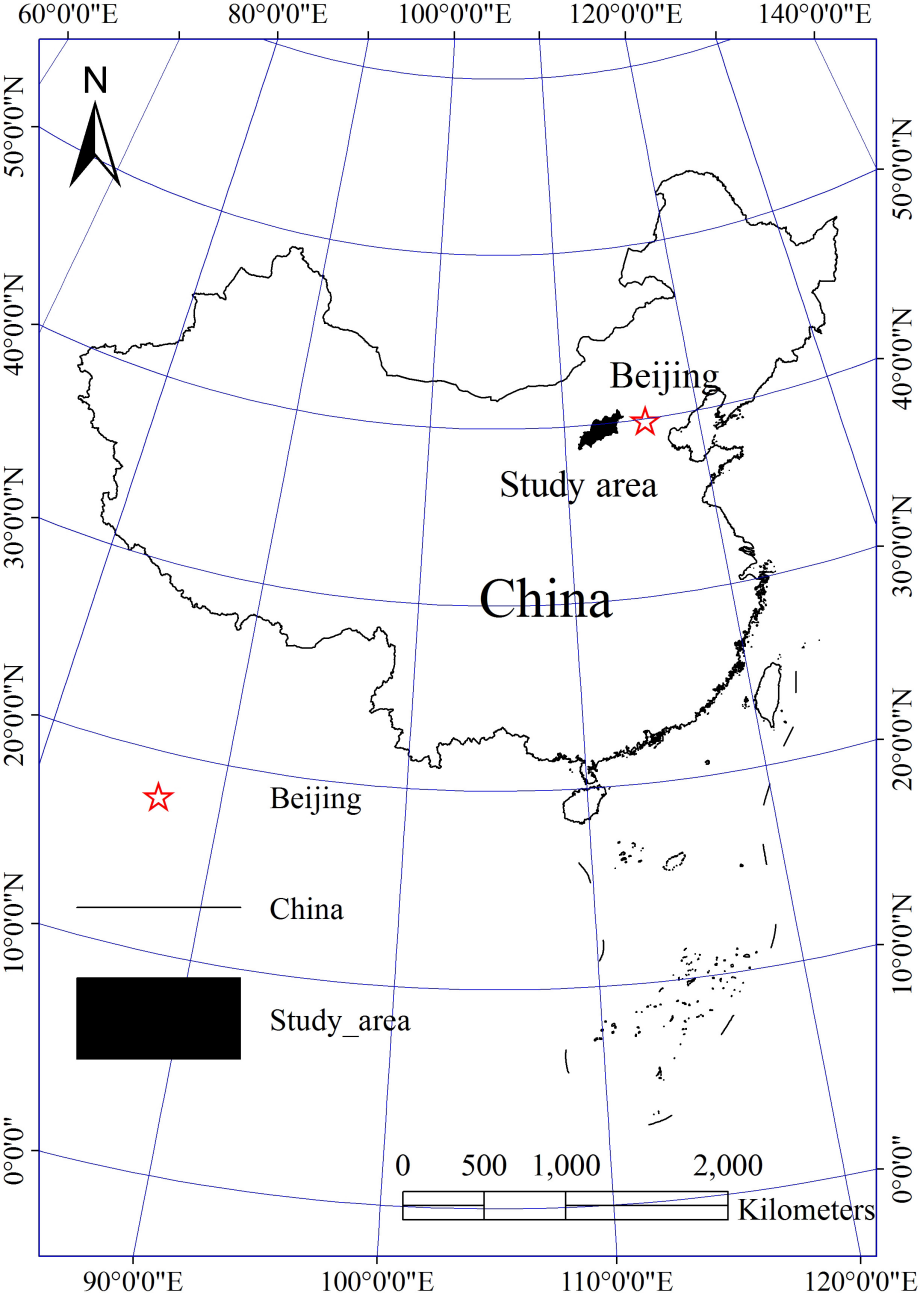
Location of the study area.

The study area is a ecologically fragile mountain and hill area with an elevation between 1200 m and 2500 m, located in the northern Shanxi province of China, roughly between 110.93°E to 114.53°E and 38.65°N to 40.72°N (Fig 1). It covers an area of about 30 000 km^2^ and includes 18 counties. The area contains no privately owned land or is protected in any way. No specific permits were required for the described field studies and the study does not involve endangered or protected species. The study area is characterized by a temperate continental monsoon climate, with dry and cold winters and arid and windy springs. The mean annual precipitation ranges from 350 mm to 500 mm, which mainly occurs during July–September. The mean annual air temperature is about 7°C, with monthly mean temperatures of −11°C in January and 21°C in July. The mean annual evaporation is approximately 2000 mm, which is nearly four times greater than the mean annual precipitation. The mean annual wind speed is more than 4.0 m s^−1^, with an average of 40 days per year of strong wind (>6.0 m s^−1^), which usually occurs from March to May. The study area is a transition zone between warm temperate deciduous forest and temperate grassland. The main vegetation types are secondary scrubs and grasses. Zonal soil mainly includes chestnut soil and cinnamon soil. The concurrence of a large number of windy days per year, low vegetation cover during winter and spring, and loose, dry surface sediments are the fundamental environmental conditions [23].

The economy of the study area is dominated by rain-fed agriculture, coal mining, and animal husbandry. During the last 10 years, the average population density has been 155.8 people per square mile, which is much higher than the population carrying capacity of 7 to 22 people per square kilometer set by the United Nations Food and Agriculture Organization for arid and semi-arid regions.

Both the physical environment and economic conditions are largely deficient. According to a bulletin of status quo of desertification and sandification in China (State Forestry Administration, P.R. China, 2011), the area of desertified land caused by wind erosion was 617 7.79 km^2^, accounting for 25.14% of the total land area in the study area. Although some large-scale national programs (e.g., Three-North Shelterbelt Program and Beijing–Tianjin Sand Source Control Program) may have had some beneficial effects on reducing dust storms and controlling desertification in our study area, aeolian desertification still remains a major environmental problem impeding local development.

### Data description

#### Proxy for desertification risk

Caused by aeolian erosion and sediment (sand and dust) deposition, aeolian desertification is frequently associated with an initial shrinkage in vegetation cover. This means that land in an unvegetated state is susceptible to desertification, and that it should be possible to determine desertification risk by evaluating the vegetation [24, 25]. Therefore, the vegetation condition of degraded land has always served as a preferred quantitative indicator of desertification at different spatial-temporal scales [26, 27].

There are a number of vegetation-cover-related variables that have proven to be valid criteria for assessing desertification risk and its evolution such as fractional vegetation cover (FVC) [28, 29], net primary productivity (NPP) [13, 30], and vegetation rain use efficiency (RUE) [31, 32]. Among these vegetation indicators, FVC can easily be interpreted from remote-sensing observations. Hence, it is treated as a proxy for ADR in our research according to China’s Land Desertification Monitoring Method (GB/T 20483–2006). Our hypothesis is that the smaller the mean annual FVC, the higher the ADR, and vice versa [16, 33].

**Fig 2:**
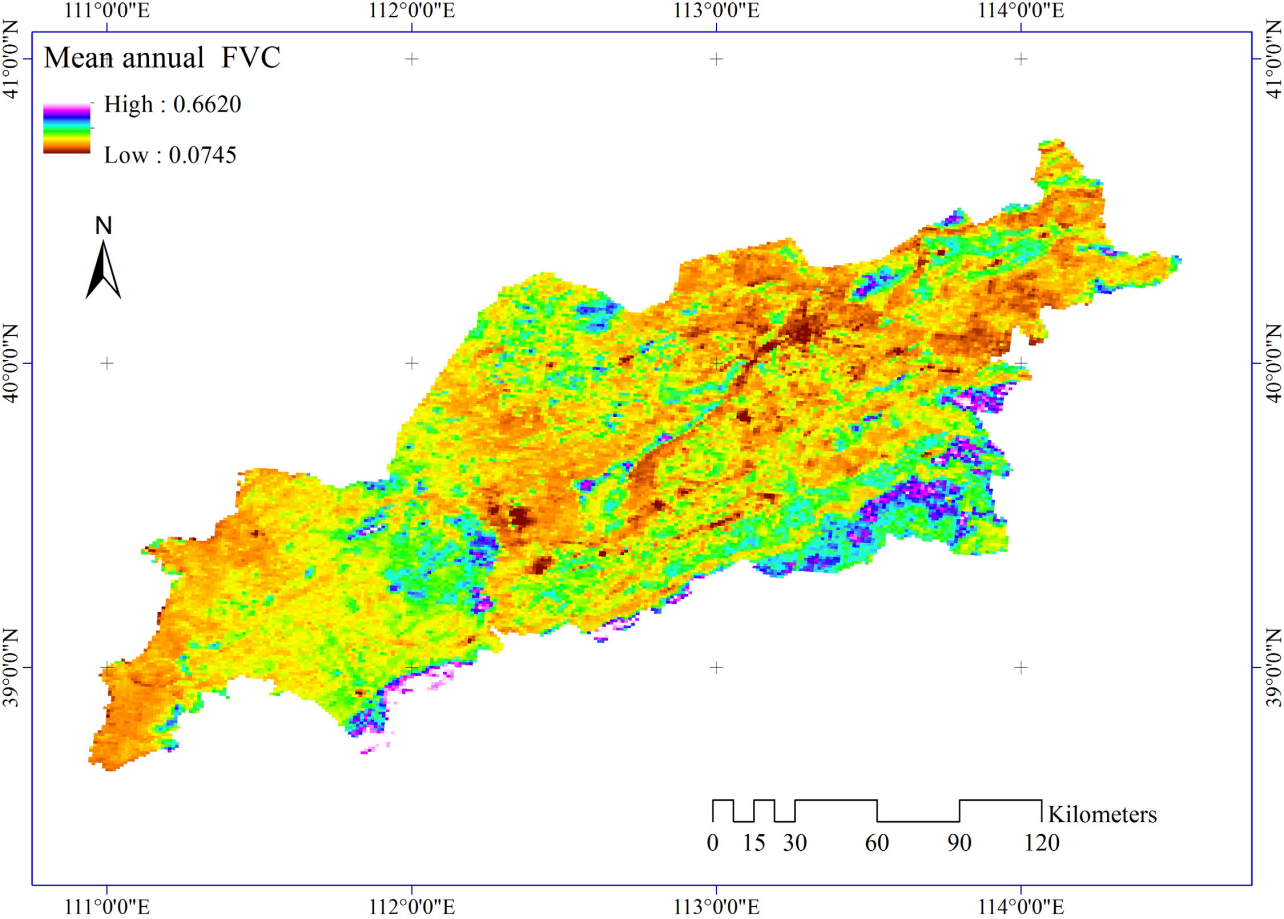
Mean annual FVC.

Monthly MOD 13A3 NDVI products obtained from the NASA LAADS (Level 1 and Atmosphere Archive and Distribution System) web with a spatial resolution of 1 km × 1 km from 2000 to 2012 were aggregated to a yearly time scale applying the maximum value compositing method [34]. Mean annual FVC was extracted using the dimidiate pixel model [35]. Fig 2 presents map of the ADR proxy. The study area was covered by four MODIS tiles (h25v04, h26v04, h26v05, and h27v05) in conjunction with 576 NDVI images. Tiled NDVI data were mosaicked, reprojected, and overlaid over the study area.

#### Determinants of desertification risk

Generally, ADR can be ascribed as the compound effect of the major determinants. Based on a review of the literature on common determinants of land degradation [5, 25, 36] and the availability of data, we examined nine variables within three main categories. Specifically, geological and physical conditions (including the following three variables: soil type, slope, and vegetation type), climatic elements (including precipitation, temperature, and wind velocity), and anthropogenic disturbances (e.g., population densityand land use type) [37, 38] were included and considered determinants of ADR in this research.

**Fig 3:**
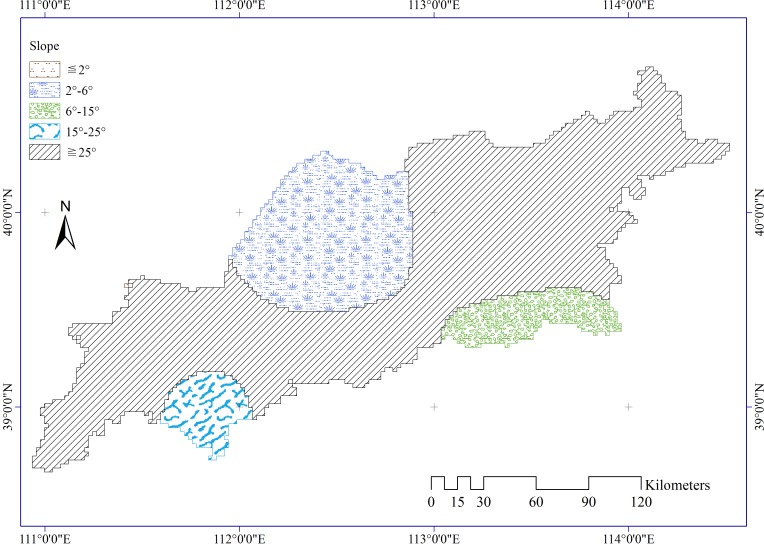
Slope distribution in the study area.

Slope (Fig 3) is defined by a plane tangent to a topographic surface, which was extracted from a digital elevation model (DEM). The 90-m DEM was derived from the NASA Shuttle Radar Topography Mission (SRTM) (http://srtm.csi.cgiar.org/SELECTION/inputCoord.asp).

**Fig 4:**
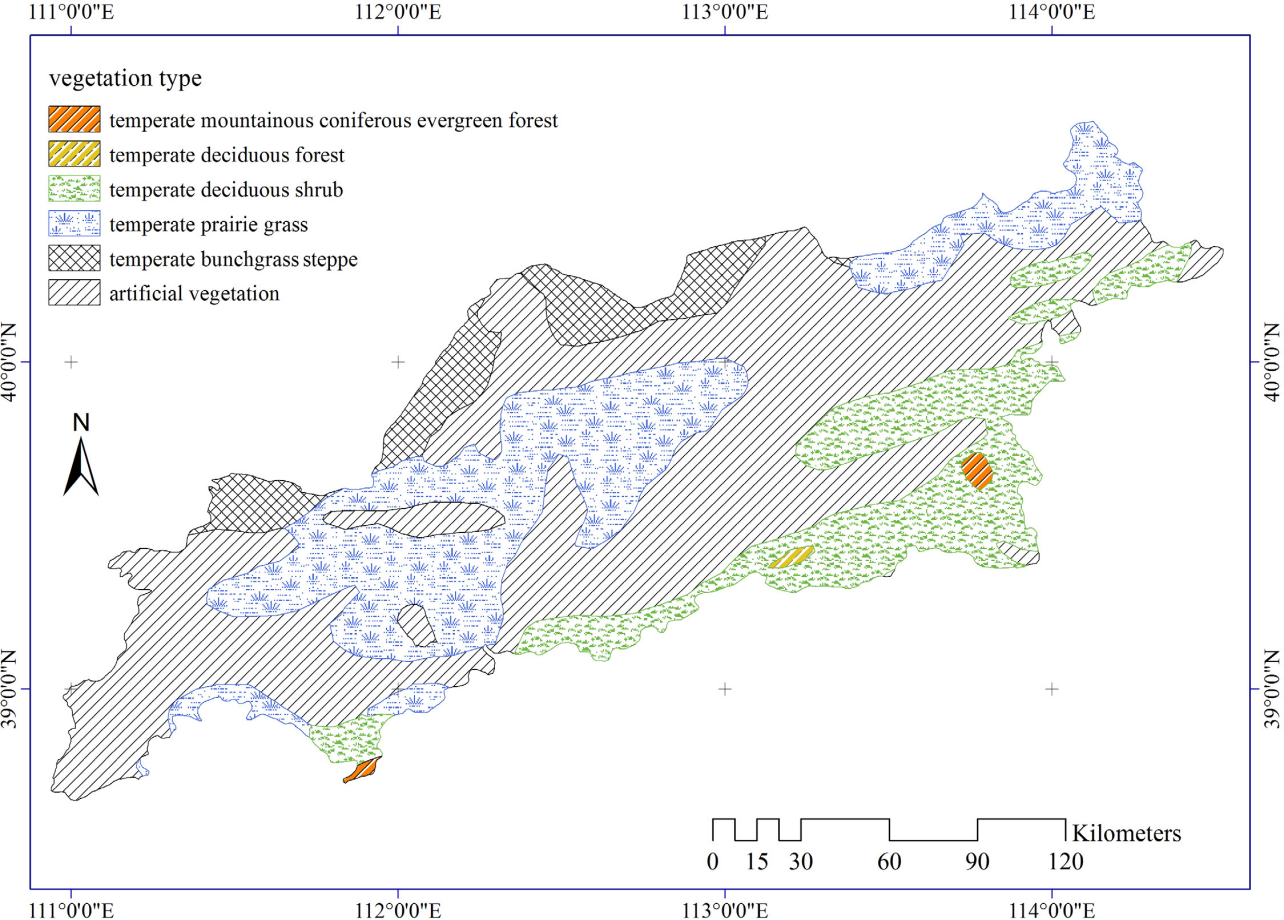
Vegetation types in the study area.

Vegetation type (VT) data (Fig 4) were obtained from the vegetation map of China (1:4,000,000), provided by the Environmental and Ecological Science Data Center for West China, National Natural Science Foundation of China (http://westdc.westgis.ac.cn).

**Fig 5:**
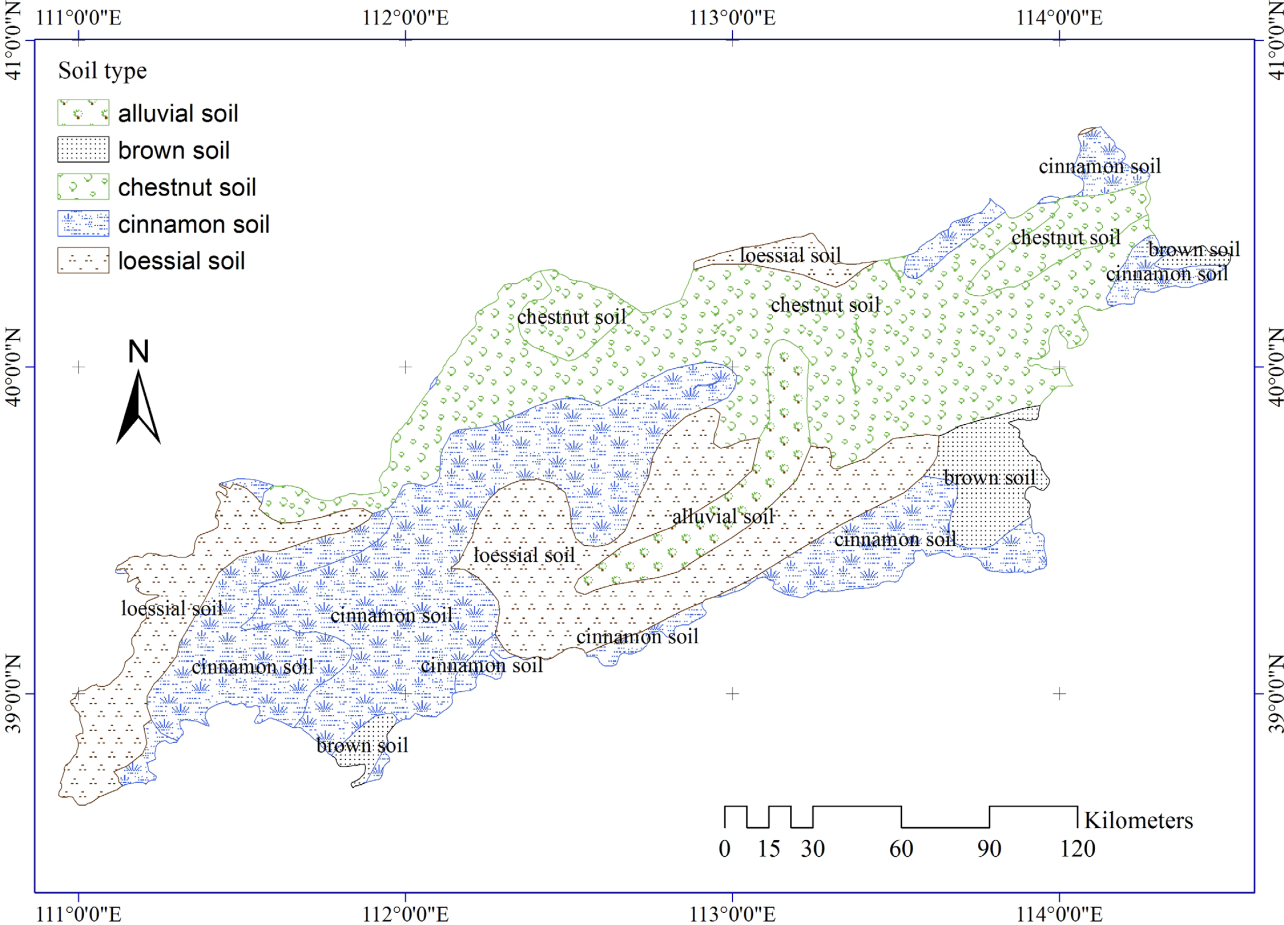
Soil types in the study area.

Soil type data (Fig 5) were extracted from the Harmonized World Soil Database, Soil Map of China (v1.1). The dataset was provided by the Cold and Arid Regions Sciences Data Center (http://westdc.westgis.ac.cn). According to soil genesis characteristics, soil types in the study area include chestnut soil, brown soil, alluvial soil, cinnamon soil, and loessial soil.

**Fig 6:**
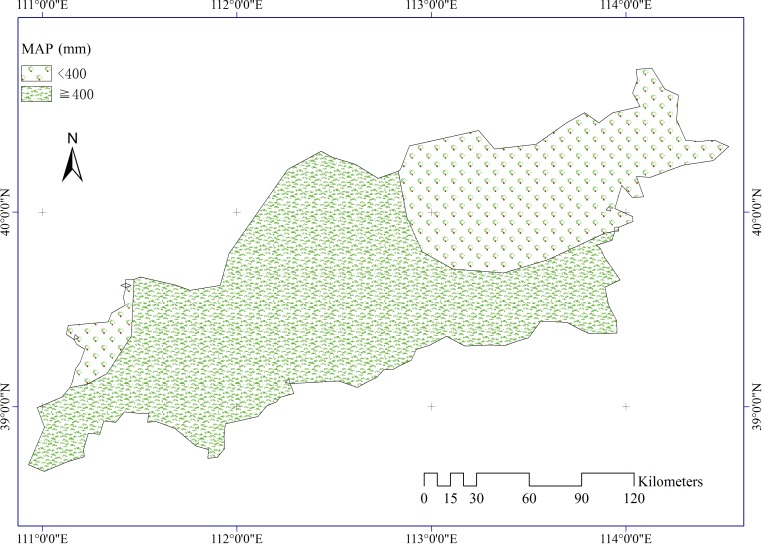
Mean annual precipitation in the study area.

**Fig 7:**
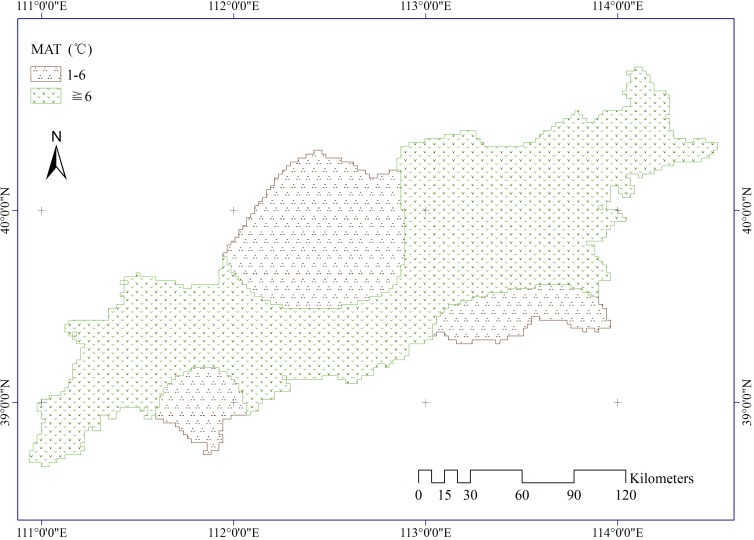
Mean annual temperature in the study area.

**Fig 8:**
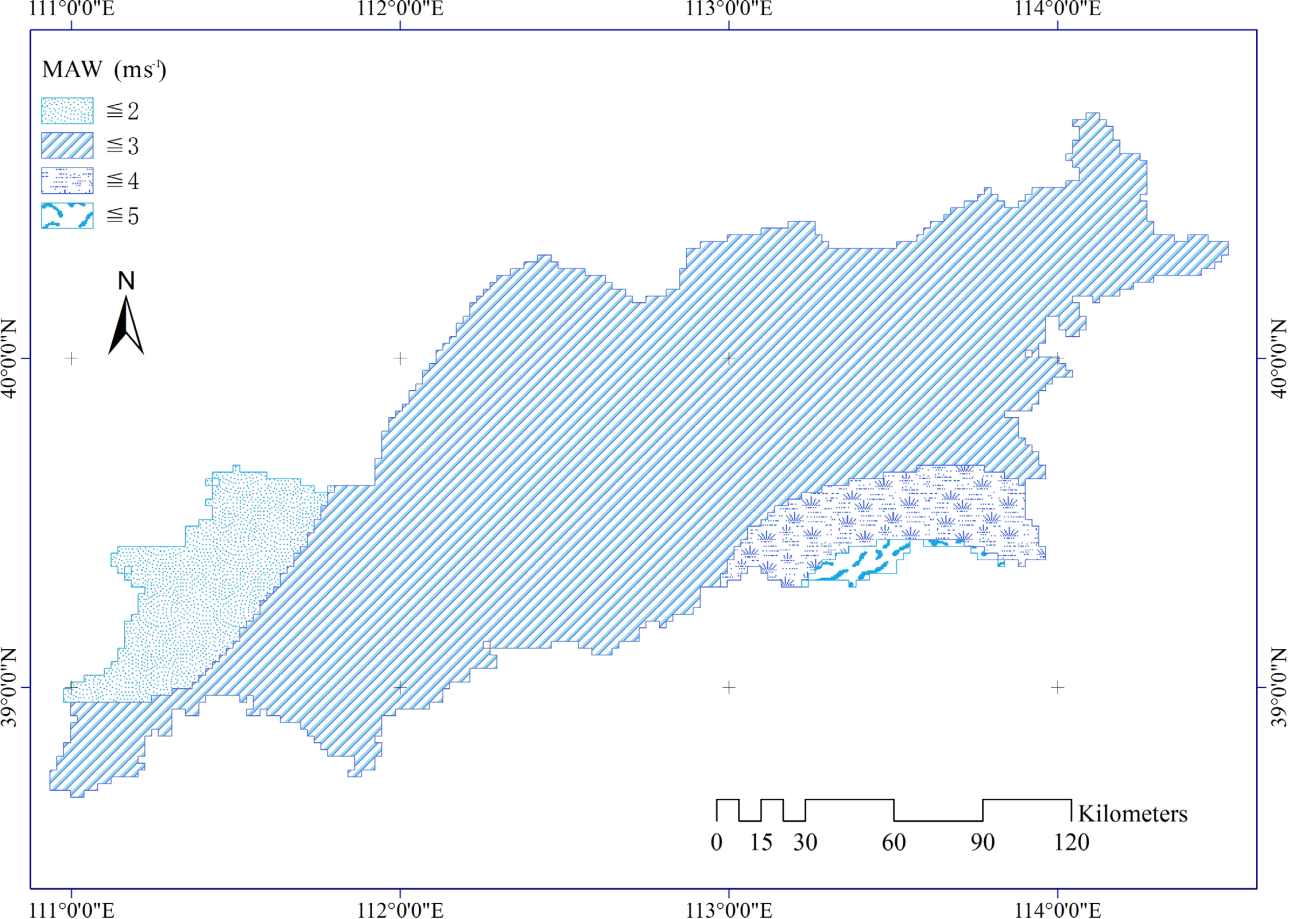
Mean annual wind velocity in the study area.

Meteorological data were collected from the China Meteorological Data Sharing Service System. There are 12 meteorological stations distributed in and near the study area. Three factors were used in our study, including mean annual precipitation (MAP, Fig 6), mean annual temperature (MAT, Fig 7), and mean annual wind velocity (MAW, Fig 8). The spatial distributions of all climatic elements were interpolated using an inverse distance weighted technique in ArcGIS software.

**Fig 9:**
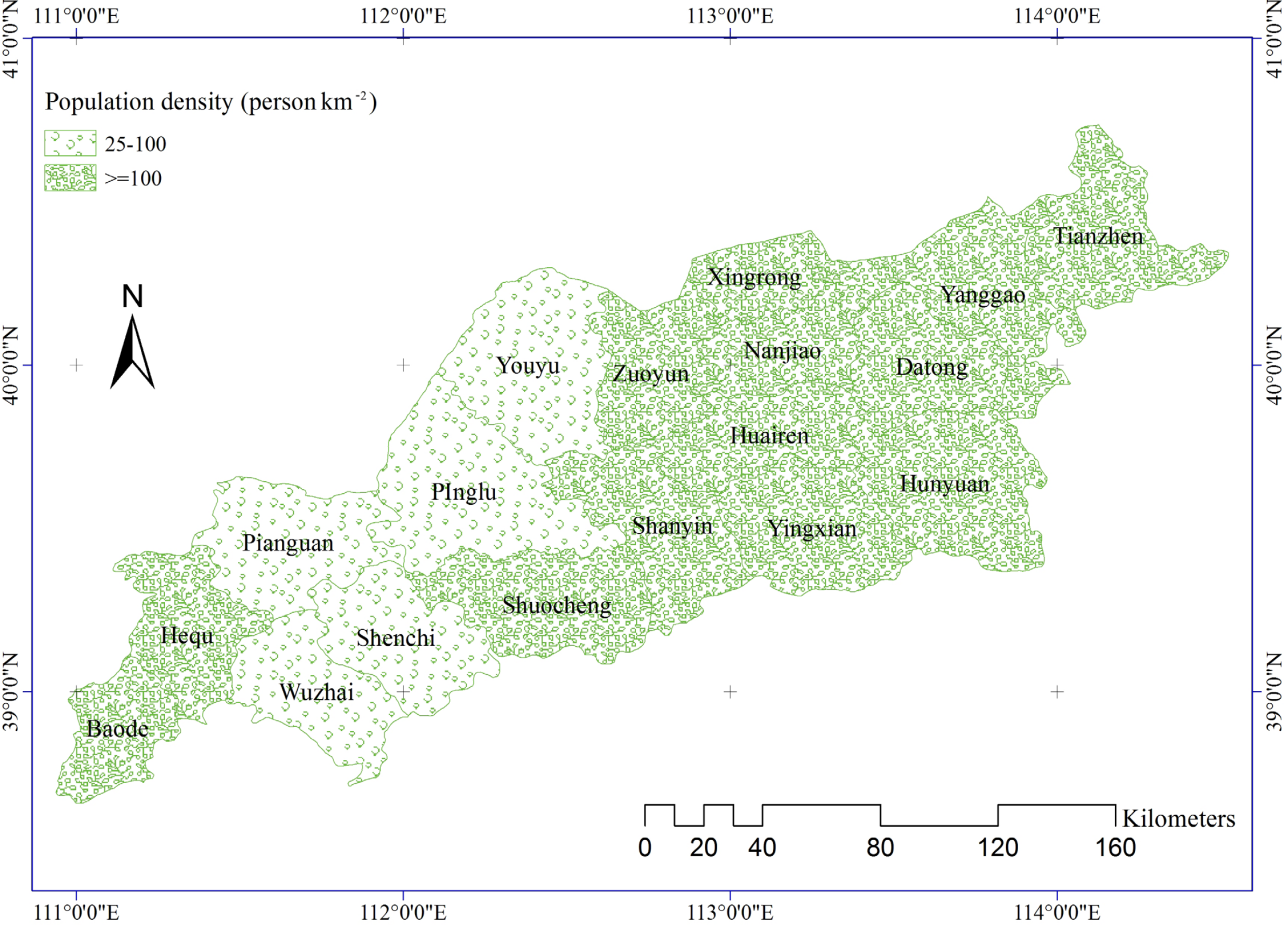
Mean annual population density.

Population data for 2000 to 2012 were collected from the Shanxi Statistical Yearbook. Population density (Fig 9) was calculated by dividing the population of each county by the area of the county.

**Fig 10:**
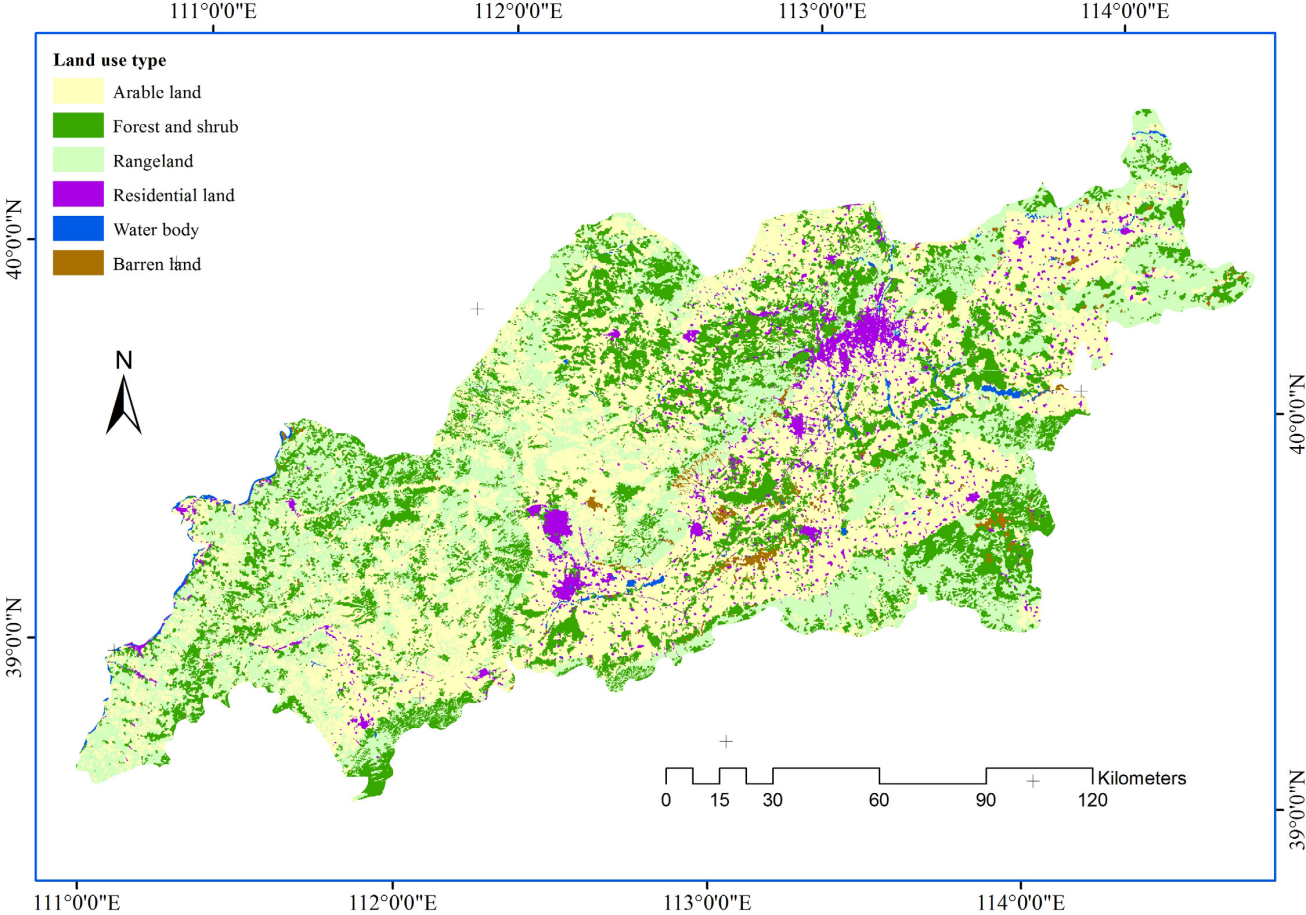
Land use in the study area.

Land use (Fig 10) was visually interpreted and digitized from Landsat TM images in ArcGIS 9.3. Land use was classified into six categories: arable land, forest and shrub, rangeland, water body, residential land, and barren land (e.g., bare land and saline land). Classification accuracy was assessed using surveys locations verified in the field in 2012. Overall accuracies were 83%. Land use data are used as a primary dataset at the local level, because they reflect the impact of human activities on land resources.

### Methods

A spatial variance analysis approach, the geographical detector model (geodetector) is used to quantitatively assess the effects of driving factors of ADR in our research. The basic principle of geodetector is to compare the spatial consistency of driving forces (e.g., geological and physical conditions, climate and anthropogenic effects) versus relevant resultant outcomes (i.e., ADR in our research) [18]. Specifically, this method assumes that a risk would exhibit a similar spatial distribution to the factors that lead to the risk [39].

The geodetector is based on the power of determinant value (PD), which is expressed by the following equation:

$$PD=1-\frac{1}{N*\sigma^{2}}\sum_{i=1}^{L}N_{i}*\sigma_{i}^{2}$$ (1)

where, *N* and *σ*^2^ denote the number of samples and the variance of risk incidence (i.e., FVC in this study) over the entire study area, respectively. The study area is stratified into *L* subregions according to the attribute of a suspected determinant or its proxy variable. *N*_*i*_ and *σ*_*i*_^2^ are the number of samples and the variance of risk incidence in the same stratum, respectively. *PD* ∈ [0,1] means that if the determinant is completely unrelated to the risk, then *PD* = 0, while if the determinant completely controls the risk, then *PD* = 1.

The geodetector can also be applied to test whether two determinants weaken or enhance each other, or whether they are independent in their contribution to the desertification risk. Therefore, the *PD* value of each determinant and the interactive *PD* value are used to quantitatively assess the relationship between potential risks and their determinants.

A full description of the geodetector can be found in Wang et al. [18, 19, 21, 39]. The geodetector used in this study is a version of Excel-GeoDetector (http://www.sssampling.org/excel-geodetector/).

All continuous data (mean annual precipitation, mean annual temperature, mean annual wind velocity, slope, and population density) were discretized into different intervals using our prior knowledge or optimal classifications methods (e.g., natural breaks etc.) [22, 40]. Then, all proxy variables from different sources were adjusted to a uniform pixel size and projection constrained by the same boundary of the study area by creating a fishnet in ArcGIS. Finally, all data derived from intersecting calculations were input into Excel-Geodetector. The above processes were performed in ArcGIS 9.3.

## Results

### Which determinants are responsible for the incidence of ADR?

We quantified the effect of determinants on observed possible risk by comparing the accumulated dispersion variance of each subarea with the dispersion variance of the entire study region. A statistical *F*-tests method was used to compare whether the accumulated variance of each subarea is significantly different from the variance of the entire study area. As described in the previous section, the *PD* value is an indicator for the contribution to the occurrence of ADR, with higher *PD* values indicating a stronger contribution.

The *PD* values of the driving factors for the incidence of ADR as represented by its assumed surrogate FVC, are in decreasing order: soil type (0.274) > mean annual precipitation (0.248) > mean annual wind velocity (0.132) > vegetation type (0.090) > slope (0.037) > population density (0.034) > mean annual temperature (0.019) > land use type (0.018). This result indicates that soil and precipitation are the major determinants that would explain the spatial distribution of the incidence of aeolian desertification risk, followed by wind velocity and vegetation type, while temperature and land use only show a weak influence. This implies that natural factors, including geological and physical conditions and climatic elements have a large relative importance for ADR in the study area.

### What are the risks of aeolian desertification for each main determinant stratum?

Comparing the differences in average risk value between subareas generated by a determinant, we searched for areas with potential risk in the determinant strata. A statistical *t-*test method was used to identify whether the average risk values among different subareas are significantly different. Bigger differences indicate greater risk within the subareas.

The average annual values of FVC for various intervals of each stratum were calculated using geodetector. The results can help discerning the differences of ADR among subareas generated by a certain driving variable. For example, the *PD* value of precipitation shows that precipitation is a critical determinant for ADR. Mean annual FVC of regions with lower MAP (<400 mm, semiarid areas) was about 49%. In contrast, regions with higher MAP (≥400 mm, semi-humid areas) had nearly 60% FVC (Table 1). In addition, the differences of mean annual FVC for both precipitation strata were significant at 95% confidence intervals (Table 2). This finding confirms the expected prediction that semiarid areas have a higher ADR than semi-humid areas, given that the latter have higher precipitation and FVC.

**Table 1:** Mean annual FVC of mean annual precipitation strata.

Stratum	<400 mm	≥400 mm
**Mean annual FVC**	48.97%	59.96%

**Table 2:** Statistical significance of both precipitation strata.

	<400 mm	≥400 mm
**<400 mm**		Y
**≥400 mm**	Y	

Note: Significant at the 5% level.

Soil type has the greatest impact on aeolian desertification according to the *PD* ranking. Aeolian desertification risk is significant different among various soil types. The order of mean annual FVC for the five soil type is: brown soil (67%) > cinnamon soil (62%) > alluvial soil (58%) > loessial soil (57%) > chestnut soil (41%) (Table 3). In addition, the differences of mean annual FVC between five soil types strata is significant at 95% confidence intervals (Table 4). This implies that each soil type has a distinct effect on the aeolian desertification risk. Areas with chestnut soil have the greatest aeolian desertification risk (lowest FVC).

**Table 3:** Mean annual FVC of soil strata.

Stratum	chestnut soil	brown soil	alluvial soil	cinnamon soil	loessial soil
**Mean annual FVC**	41.43%	66.95%	57.72%	61.92%	56.93%

**Table 4:** Statistical significance of soil strata.

	chestnut soil	brown soil	alluvial soil	cinnamon soil
brown soil	Y			
alluvial soil	Y	Y		
cinnamon soil	Y	Y	Y	
loessial soil	Y	Y	Y	Y

Note: Significant at the 5% level.

Similar analyses were performed to investigate associations between other variables and ADR to find areas of potential risk for each stratum and attributed variable.

### Are the potential determinants of ADR independent or dependent of each other?

We analyzed the effect of the interaction (symbolized by ∩) of two or multiple determinants of ADR by comparing the combined contribution of two individual determinants to a risk as well as their independent contributions.

The combined effects of geological, physical, and climatic determinants are summarized in Table 5. The results are shown as interactive *PD* values. All interactive *PD* values are greater than the highest *PD* value of a single factor (i.e., the *PD* value of soil type, which is 0.274). Furthermore, interactive effect of precipitation combined with vegetation type shows a non-linearly enhanced impact (defined as the interactive *PD* value of two variables being higher than the sum of the two individual *PD* values). These findings indicate that the interplay between determinants plays an important role for potential aeolian desertification.

**Table 5:** *PD* values for interactions between geological, physical, and climatic determinants.

	Soil type	Mean annual precipitation	Slope	Vegetation type	Mean annual temperature
**Mean annual precipitation**	0.394				
**Slope**	0.325	0.280			
**Vegetation type**	0.339	0.358^*^	0.122		
**Mean annual temperature**	0.228	0.258	0.067^*^	0.133^*^	
**Mean annual wind velocity**	0.351	0.307	0.123	0.136	0.088

*non-linearly enhanced effect (i.e., *PD* (A∩B) > *PD* (A) + *PD* (B)).

The effect of anthropogenic influences combined with geological and physical variables on the occurrence of ADR was also investigated (Table 6). All interactive *PD* values are higher than any *PD* value of a single impact variable. In addition, anthropogenic factors combined with geological and physical variables exhibit a non-linearly enhanced impact on ADR.

**Table 6:** *PD* values for interactions between geological/physical and anthropogenic determinants.

	Population density	Land use type
**Soil type**	0.335*	0.292
**Vegetation type**	0.180*	0.127*
**Slope**	0.084*	0.075*

*non-linearly enhanced effect (i.e., *PD* (A∩B) > *PD* (A) + *PD* (B)).

In comparison to geological and physical determinants, interactions between human activities and climate seem to be much more complicated (Table 7). For example, population density and wind velocity are recognized as a non-linearly enhanced impact on ADR (pop∩Wind = 0.159 > pop (0.034) + Wind (0.085)). The same relationship is observed between population density and precipitation. Moreover, there are other joint impacts between two determinants such as the combination of population density with temperature (pop∩Temp = 0.053 = pop (0.034) + Temp (0.019)), land use type with wind velocity (Land use∩Wind = 0.103 = Land use (0.018) + Wind (0.085)), and temperature with land use type (Temp∩Land use = 0.037 = Temp (0.019) + Land use (0.018)). The combination of these factors did not increase the *PD* values but rather implies that they are independent of each other in driving ADR, which is defined as an independent effect.

**Table 7:** *PD* values for interactions between climatic and anthropogenic determinants.

	Population density	Land use type
**Mean annual precipitation**	0.250	0.259
**Mean annual temperature**	0.053˜	0.036˜
**Mean annual wind velocity**	0.159*	0.102˜

*Non-linear enhanced effect (i.e., PD (A∩B) > PD (A) + PD (B)); ˜independent effect (i.e., PD (A∩B) = PD (A) + PD (B))

## Discussion

There are several natural and anthropogenic driving forces with large impacts and relative importance. We found that soil type, precipitation, and wind velocity are mainly responsible for ADR, which indicated an erodible soil integrate with an erosive climatic conditions were very prone to trigger a high ADR. The interactive effects between pairs of above determinants are greater than their individual impacts. Although the individual effects of slope, population size, temperature, and land use type are weak, they contribute more strongly to ADR when combined with soil type, precipitation, wind velocity and vegetation type.

Previous research indicated soil types played an important role in speeding up the desertification process[41]. We also found that soil type has the greatest effect on ADR. The dominant soil types in the study area are chestnut soil, brown soil, alluvial soil, cinnamon soil, and loessial soil. The parent material for these soils originated from loose Quaternary sediments, which are highly vulnerable to wind erosion due to an abundance of unconsolidated sand grains. As a result, these soil types are highly prone to degradation [3].

Climate affects aeolian desertification mainly through changes in air temperature, precipitation, and wind velocity [42]. The *PD* results illustrate that precipitation and wind have a greater effect on the occurrence of aeolian desertification than temperature. According to the data for our study area, annual and seasonal mean precipitation has shown a fluctuating increase over the past decades, and annual and seasonal mean wind velocities have varied with a clearly decreasing gradient. Especially in spring and winter, ADR is high because of low vegetation cover. The increase in precipitation activated the vigor of surface vegetation, and the decrease in wind velocity benefited soil and water conservation. Therefore, changes in precipitation and wind activity have contributed to a reduction in aeolian desertification areas as identified by FVC. Annual and seasonal mean temperature obviously increase. The increasing temperature would cause an increase in FVC, Thus the desertified areas which was defined by FVC reduced. However, increased temperature should increase topsoil evaporation and reduced topsoil moisture content. Consequently, ADR increased correspondingly. The continuous warming was consistent with the decrease of aeolian desertification in recent decades years in our study area. One possible reason is that increasing precipitation and decreasing wind velocity maybe offset the adverse effect of temperature on land desertification (e.g., *PD* (precipitation∩temperature) = 0.258 > *PD* (temperature) = 0.019, and *PD* (wind velocity∩temperature) = 0.088 > *PD* (temperature) = 0.019). Although anthropogenic activities and climate fluctuations have jointly caused desertification in semi-arid North China [11], the findings of our study highlight the importance of geological, physical, and climatic elements for accelerating the incidence of ADR. For instance, *PD* values demonstrate that the impact of human activities on ADR is much lower than that of natural factors. This means that the proxies we used for human activities did not support the hypothesis that anthropogenic factors are principally responsible for ADR. These results are in contrast to conclusions by previous studies [3, 11] of semi-arid China that human activity is a contributing factor to desertification in this region. From 1999 to 2011, the population in the region has risen to almost three times the original level. In contrast, the desertified area decreased by 1,658.830 km^2^ from 7,836.64 to 6,17.790 km^2^ during the same period in our study area according to a bulletin of status quo of desertification and sandification in China. Evidently, the decrease in area of desertified land is inconsistent with the rapid increase in population. The population density showed a relatively weak effect to population on the incidence of ADR according to the *PD* value. One possible One possible reason is that we used the total population rather than population density in rural areas as the variable. However, the population density in rural areas data was not available in our study area. Similarly, landuse had a weak influence on the ADR. One plausible explanation is that the yearly-scale landuse data could not precisely match the FVC which was the mean value of 13 years.

Although we used a new spatial variance analysis method to compare spatial consistency of risk distribution versus the determinant strata, there are some limitations to our study. For example, the divergence interval of discretize continuous parameters and the lagged effect of determinants on FVC might be impact the results. Consequently, the presented exemplary case highlights that the effect of the determinants of land desertification risk is not likely to be common in all terrestrial zones. Despite the above-mentioned issues, the results obtained in our preliminary analysis are valuable when proposing policies to control land desertification. In the future, we intend to improve our understanding of the causes for desertification by applying the knowledge gained in this study and conducting long-term field observations.

## Conclusions

In this study, a geographical detector model and GIS techniques were applied to assess the effects of geological, physical, and climatic elements as well as human activities on ADR. Interrelationships between the ADR and its determinants were extracted by studying the correspondence of their spatial distribution. The order of the *PD* values of single determinants was used to identify their relative importance for the incidence of ADR, and interactive *PD* values were utilized to investigate the combined impact of determinants.

The unique contribution of this study is that we not only assessed the relative role of determinants triggering ADR, but also estimated the effects of the interaction of different determinants, which was lacking in previous studies.

The results demonstrate that soil type, precipitation, and wind speed have the greatest effect on ADR. Our results also imply that geological and physical elements (e.g., soil attributes) and climatic factors (e.g., precipitation and wind) rather than human activities play a greater role in the incidence of ADR in our study area of northern China. Furthermore, the interaction of certain driving forces lead to non-linearly enhanced impacts on ADR. These findings will assist local inhabitants and policy makers when developing measures for wind prevention and sand control (e.g., planting windbreaks and forest belts, improving soil and water management, expansion of irrigation, rural-urban migration, grain for green project, et al) to mitigate the effects of desertification.
